# Medical and Philosophical Intelligence

**Published:** 1811-01

**Authors:** 


### MEDICAL AND PHILOSOPHICAL INTELLIGENCE.

### ROYAL SOCIETY.

On the 22d of November, Dr. Wollaston read a paper "On some of the Combinations of oxymuriatic Gas and Oxygen, and on the Chemical Relations of these Principles to inflammable Bodies." By H. Davy, Esq Sec. R. S.

In this paper Mr. Davy details a great number of experiments which he has made on the combinations of oxymuriatic gas and oxygen with the metals of the fixed alkalies, the metals of the earths, and the common metals; with a view to illustrate the nature, properties, and combinations of oxymuriatic gas, and its relations to inflammable bodies, as compared with those of oxygen. He also offers some general views and conclusions concerning the chemical powers of different species of matter, and the proportions in which they combine. And lastly, he concludes his paper by some observations on the impropriety of the present nomenclature, in reference to the oxymuriatic gas and its combinations; and proposes some concise modes of distinguishing these novel bodies.

Mr. Davy made some previous experiments on the combinations of potassium and sodium with oxygen; and of potash and soda with water, from which he concludes that those metals when burnt with oxygen gas are at their highest state of oxygenation—and at their lowest, when in the state of potash and soda. He also found that ignited potash contains about 16 per cent. of water, and ignited soda 22-9 per cent.

The spontaneous inflammation of the metals of the fixed alkalies in oxymuriatic gas, affords a proof of the intensity of their attractions. In these operations, no water is separated, but mere binary combinations formed; the same as those produced by igniting muriate of potash and soda. Similar compounds are formed when dry potash and soda are heated in oxymuriatic gas, and oxygen is evolved.

Mr. Davy mentions a simple mode by which pure sodium may be obtained. It is by mixing common salt which has been ignited to redness, with potassinm, and exposing the whole to a red heat in a glass tube or retort: for every two parts of potassium employed, one part of sodium is obtained.

As the muriates of lime, barytes and strontites remain unaltered by any simple attractions, even at a white heat, Mr. Davy conceived that these compounds consist merely of the metallic bases of the earths in union with oxymuriatic gas, and the experiments he has made confirm this conclusion. Thus when lime, barytes, &c. were heated in oxymuriatic gas, oxygen was expelled, and substances exactly similar to the dry muriates were formed.

In operating on metals, Mr. Davy employed green glass retorts holding from three to six cubical inches of gas, they were furnished with stop-cocks, The metal was first introduced into the retort, it was then exhausted and filled with oxymuriatic gas, the heat of a spirit lamp was employed in the processes. The products from arsenic, antimony, and bismuth; and on the addition of water, the white oxides and muriatic acid. Tin produced Libavius's liquor, mercury, corrosive sublimate, silver silver and lead, horn silver, and horn lead. Iron, a beautiful, volatile, crystallized substance which gave the red muriate of iron on the addition of water.

Mr. Davy also found that oxymuriatic gas decomposes the metallic oxides at a heat below redness: those of the volatile metals more easily than those of the fixed metals, and protoxides more readily than peroxides. Mr. Davy notices two beautiful experiments on the agency of oxymuriatic gas on white oxide of arsenic, and black oxide of iron. In these cases, no oxygen was evolved, the portion separated from one part of the oxides combined with the other part, and the products were butter of arsenic and arsenic acid, and ferruginous sublimate and red oxide of iron.

Mr. Davy notices an experiment in which he decomposed the gray oxide of tin by muriatic acid gas. In this case, water rapidly separated and Libavius's liquor was formed.

Mr. Davy conceives that these new inquiries confirm all the conclusions he has drawn in his recent paper on "Oxymuriatic Acid, &c."

---

With a liberality consonant to the high station in which they are placed, the President and Fellows of the College of Physicians of London, have determined on printing their *Statutes*; and by that means, making distinctly known to the public the extent and nature of their powers.

---

A case of the ancient *Elephantiasis, or Lepra Arabum*, has occurred at Bartholomew's Hospital, exactly answering Dr. Adams's description of that dreadful disease. The countenance has a near resemblance to one of his plates. The subject is a Portuguese Brazilian, about thirty years old, has little hair on the chin, none on the pubes, and in all other respects, answers the history of the disease as given in the "Observations on Morbid Poison"

---

*Medical Spring in Lancashire.*

About two miles from Flookborough, a small village in the parish of Cartmell, in Lancashire, near to a very ancient building, *Wroryshölme Tower*, there is a medicinal spring, called, according to common usage, Holywell. From the bottom of a rock adjoining to the *Tower*, the water appears to issue, and is sold at a very cheap rate, by a person residing in a hut, who is little acquainted with the qualities it possesses, to those afflicted with scurvy, or any cutaneous disease. As this spring is stated to be little known, we shall be indebted to any gentleman of Cartmell, or the neighbourhood, who will give a more particular account. Is it of the nature of the Borrodale, and Rougham, or New Cartmel Springs? Scurvy is used incorrectly, we apprehend, for some of the species of Herpes, or other individual of the numerous tribe of cutaneous maladies.

---

The scarcity of oil at Venice, in 1807, occasioned by the destruction of the olive trees, during the war, led to the introduction into that state of of the Chinese radish, which has, of late, been cultivated there with great success. The oil of this plant is represented to be superior to any yet known, not merely for the table, but for burning, and many medical purposes.

---

*Mr. Stevenson* has made some important improvements in the treatment of *blear-eye*, by which he is enabled to effect a very speedy and radical cure, with scarcely any pain to the patient. We hope soon to have an opportunity of stating the particulars of this improvement in the treatment of this obstinate and distressing disease.

---

*Test for the Discovery of Sulphuric Acid in Vinegar.*

The Acetite of Barytes is a most delicate test for the detection of this sophistication. Upon the application of a small quantity of this acetite to vinegar thus adulterated, a copious insoluble sediment is immediately precipitated.

---

*Mr. James Ward*, an eminent artist, residing in Newman-street, is engaged in etching the portraits of Mary Thomas, the fasting woman of North Wales; and of Ann Moor, of Tutbury, who has likewise lived a long time without taking nutriment. These portraits will be published early in the spring, and are now in sufficient forwardness, to manifest all that spirit and freedom which renders the etching of painters so peculiarly valuable. Notices of these two women, so remarkable by the circumstance of their continuing to exist for so long a time, literally without food, will be found in the preceding volumes of this Journal. Mr. Ward proposes to publish with these portraits, the fac simile of a letter written during sleep. Connected with this letter, which was written by a person named Bush, will be given some surprising facts respecting the operation of the intellectual principle during the period of sleep; and which add considerably to the stock of materials that at present constitute the history of somnambulism.

---

*Charles Wilkinson, Esq.* after a series of observations, made during several years, on the Influence which the Moon has upon the Atmosphere of this Earth, gives the result of those observations in the following Table, the calculations in which, we are assured by him, are sufficiently near to the truth, to ascertain, generally, what weather will follow the times of the changes in that Planet, as there specified. The effect resulting from every individual change in the Moon, occupies the space of time up to the next change, so that each prognostication continues in force for eight days. If a more extended observation, and the experience of successive years should support these conclusions, great benefit will arise to Agriculture, various rural employments, and maritime affairs.

*TABLE.*

### TABLE.

| MOON. | SUMMER. | WINTER. |
|---|---|---|
| If it be New or Full Moon, or the Moon enters into the First or Last Quarter at the hour of 12 at Noon;—or between the Hours of | Very Rainy | Snow or Rain |
| 2 — and — 4 | Changeable | Fair & Mild |
| 4 — 6 | Fair | Fair |
| 6 — 8 | Fair if W. at N. W. Rainy if S. or S. W. | Fair and Frosty if N. or N. E.—Rain or Snow, if S. or S. E. |
| 8 — 10 | Same | Same |
| 10 — Midnight | Fair | Fair & Frosty |
| Midnight — 2 | Fair | Hard Frost, unless W. at S. or W. |
| 2 — | Cold with freq. showers | Snow & Stormy |
| 4 — 6 | Rain | Cold Rain or Snow |
| 6 — 8 | Wind & Rain | Stormy |
| 8 — 10 | Changeable | Cold Rain if W. at W.—Snow, if E. |
| 10 — 12 | Frequent Showers | Cold with high wind |

### *Migration of Swallows.*

The disappearance of the Swallow Tribe in the Autumn has always excited the attention of naturalists. Various hypotheses have been formed to account for this fact. That they either migrate from, or hibernate in this country, is certain: but which of the two still divides opinion.

Mr. Foster has lately printed (*Phil. Mag. October* 1810) some observations on the hirundines of this country, in favour of migration.

The Swift (*Hirundo apus Lin. Martinet noir, Buff.*) which abounds, he says, in the neighbourhood of Hackney, and annually builds in great numbers in the old steeple, was not seen after the 13th of August: several days after this (on the 17th) Mr. Foster being at Ely, saw great numbers of this kind flying round the tower of the cathedral; after which not a singel bird was seen.

In consequence of the controversy so long carried on among naturalists, whether the swallow was a bird of passage, or whether it remained dormant, during the winter, Mr. Foster opened several holes of the sand martin (*Hirundo riparia, Lin.—L'Hirondelle de rivage, Buff.*) but found nothing in them but toads, which had there taken up their winter lodging. There are many circumstances which tend greatly to establish the opinion, that these birds migrate: for instance, they do not appear in spring and depart in autumn sooner or later according to the the forwardness or backwardness of the season, but generally according to the direction of the prevailing current of air. The prevalence of N. E. winds occasioned a very great diminution of the numbers of swallows and martins, this year, before their usual time; whence it may be inferred that they had taken their flight in a south-western direction. The winged insects (the food of this tribe) were very abundant, when this migration took place. Many proofs that swallows hybernate in the countries where they have been seen in the summer, are to be gathered from the ancients. Olaus Magnus and Scheffer, in a letter to Hevelius, affirm, that, in some northerly countries, it is very common for fishermen to find in the water large clusters of swallows, which in the beginning of the Autumn had thus piled themselves up. Kircher, in Mund. Subterran. lib. 8. Sect. 4. observes, that some Swallows migrate, whilst those in cold countries conceal themselves in caves, lakes, hollow trees, &c. Fortunus Licetus affirms, that the Swallows which in Summer build their nests on the tops of houses towards the end of September plunge into ditches and rivers, and remain till the middle of May. Huet, archbishop of Avranches, informs us, that between Caen and the sea, along the shore of the river Orne, large clusters of swallows are found in the Winter suspended in the caves like bunches of grapes. Pedo Albinovanus, in his elegy on the death of Mecænas, speaks of the retreat of the Swallows to the rocks, as an infallible sign of the approach of winter.

"Congelantur aquæ, scopulis se condit hirundo
Verberat e gelidas garrula vere lacus."

Various other writers might be quoted in support of these facts.

---

The following intelligence from Dublin, from unquestionable authority, proves that the state of medical education in the sister kingdom is rapidly advancing.

"The College of Surgeons is now completed, and will, when some proposed additions are made to the anatomical department, excell any thing of the kind in the Empire. The present dissecting room is inadequate to accommodate a class which, last season, amounted to *more* than three hundred students, and which has been rapidly increasing for four years past. The facility with which dead bodies are procured here, and the indifference with which all classes of the people look upon the business of dissection, give us advantages which, as far as I can learn, are not enjoyed to the same extent in the schools of either London or Edinburgh, so that we have every reason to believe that Dublin, in a few years, will have established a character of respectability for Anatomical Instruction.

The School of Physic too in this city, is likely to become conspicuous. A very magnificent Clinical Hospital is now almost finished, which will be a great acquisition to the Students, and will enable our own countrymen to graduate here, without going to the expence and inconvenience of a journey to Edinburgh."

---

*Mr. George Graves*, nephew of the late ingenious and accurate Botanist, *William Curtis*, is engaged in a work on British Ornithology. It is intended to publish this work in numbers, each of which is to contain six coloured Engravings. Copious descriptions will be given of the peculiar manners of each species of British Birds, the modes of nidification, generic and specific character, with other particulars, illustrative of their habits. Such birds as have been domesticated, though originally foreign, will be placed in an Appendix, making a part of the fourth or last volume. The work is in such forwardness as to insure an immediate publication of the first number.

When we are informed that the plan of the work was projected by the scientific Author of the Flora Londinensis, and that many of the Drawings were executed under his care, and that Mr. Graves is actuated by the same ardent desire to improve Natural History, that his late relation possessed, we cannot doubt, but this work will be highly acceptable to the Ornithologist.

---

*Anatomical Theatre*, Bristol. Mr. Shute will commence his Spring course of Lectures on Anatomy, Physiology, and the principles of, and operations in Surgery, on Saturday the 12th of January, at eight o'clock in the morning. Park-street, December 21st, 1810.

---

Dr. Buxton will commence his Spring Course of Lectures on the Practice of Medicine, about the 20th of January, 1811, at the London Hospital.

---

Lectures on Anatomy, Physiology, Pathology, and Surgery, by Mr. John Taunton, Member of the Royal College of Surgeons of London, Surgeon to the City and Finsbury Dispensaries, City Truss Society, &c.

In this Course of Lectures it is proposed to take a comprehensive view of the structure and œconomy of the living body, and to consider the causes, symptoms, nature, and treatment of surgical diseases, with the mode of performing the different surgical operations; forming a complete course of anatomical and physiological instruction for the medical or surgical student, the artist, the professional or private gentleman.

An ample field for professional edification will be afforded by the opportunity which pupils may have of attending the clinical and other practice of both the City and Finsbury Dispensaries.

The Spring course will commence on Saturday, January the 12th, 1811, at eight o'clock in the evening, *precisely*, and to be continued very Tuesday, Thursday, and Saturday, at the same hour.

Particulars may be had on applying to Mr. Taunton, Greville-street, Hatton Garden.

---

Theatre of Anatomy, Blenheim-street, Great Marlborough-street. The Spring Course of Lectures on Anatomy, Physiology, and Surgery, will be commenced on Monday, the 21st of January, at two o'clock, by Mr. Brookes.

Spacious Apartments, thoroughly ventilated, and replete with every convenience, are open all the Morning for the purposes of

Dissecting

Dissecting and Injecting, where Mr. Brookes attends to direct the Students, and demonstrate the various parts as they appear on Dissection.

---

In the beginning of January Dr. Adams will commence his Course of Lectures on the Institutes and Practice of Medicine at his house, No. 17, Hatton Garden; to be continued every Tuesday, Thursday, and Saturday, at ten o'clock in the morning.

---

Mr. Joseph Murphy, surgeon, in a few days will publish a Natural History of the human Teeth, with a treatise on their Diseases from infancy to old age, adapted for general information: to which are added Observations on the Physiognomy of the Teeth and projecting Chin.

---

*The following Letter was received too late to be inserted in its proper place.*

GENTLEMEN,

IN the life of Dr. Beddoes, lately published by me, an accidental error has been detected, which I should be happy to avail myself of the medium of your journal to correct.

From the account given at page 389, it would appear as if Dr. Craufuird had expressed a wish that further advice should be called in, when the alarming change had already taken place, which so shortly preceded Dr. Beddoes's dissolution. The fact, however, is, as I have since been informed, that this wish was expressed, not by Dr. Craufuird, but by some members of the family, and though complied with on his part, was accompanied by a remark that it must necessarily be useless.

*Bristol, Dec.* 1810.

I remain, Gentlemen,
Your very humble Servant,
J. E. STOCK.

